# Accelerating structural dynamics through integrated research informatics

**DOI:** 10.1063/4.0000759

**Published:** 2025-07-30

**Authors:** Ben Eisenbraun, Alex Ho, Peter A. Meyer, Piotr Sliz

**Affiliations:** 1 Department of Biological Chemistry and Molecular Pharmacology, Harvard Medical School, Boston, Massachusetts 02115, USA; 2 Department of Pediatrics, Boston Children's Hospital, Boston, Massachusetts 02115, USA

## Abstract

Structural dynamics research requires robust computational methods, reliable software, accessible data, and scalable infrastructure. Managing these components is complex and directly affects reproducibility and efficiency. The SBGrid Consortium addresses these challenges through a three-pillar approach that encompasses Software, Data, and Infrastructure, designed to foster a consistent and rigorous computational environment. At the core is the SBGrid software collection (>620 curated applications), supported by the Capsules Software Execution Environment, which ensures conflict-free, version-controlled execution. The SBGrid Data Bank supports open science by enabling the publication of primary experimental data. SBCloud, a fully managed cloud computing platform, provides scalable, on-demand infrastructure optimized for structural biology workloads. Together, they reduce computational friction, enabling researchers to focus on interpreting time-resolved data, modeling structural transitions, and managing large simulation datasets for advancing structural dynamics. This integrated platform delivers a reliable and accessible foundation for computationally intensive research across diverse scientific fields sharing common computational methods.

## I. INTRODUCTION

### A. The growing importance and challenges of structural dynamics

Understanding the dynamic nature of molecular systems is fundamental to deciphering mechanisms in biology, chemistry, and materials science. Structural dynamics focuses on characterizing the changes in electronic and geometric structures over time, revealing insights into function, reaction pathways, and conformational landscapes that static structures alone cannot provide. Experiment techniques, such as time-resolved x-ray crystallography and cryo-electron microscopy (cryoEM), and purely computational methods, such as molecular dynamics (MD) simulations and integrative/hybrid approaches making use of complementary techniques, all increasingly supported by sophisticated artificial intelligence/machine learning (AI/ML) approaches, are pushing the frontiers of the field. These methods allow researchers to probe transient states, visualize molecular motion, map energy landscapes, and model complex biological processes like enzyme catalysis with unprecedented detail. However, these advanced techniques can generate vast amounts of data and demand significant computational resources, posing substantial challenges in data processing, analysis, and interpretation; likewise, integrative/hybrid approaches have the potential for increasing data and workflow complexity. For instance, large-scale MD simulations aiming to capture biologically relevant timescales, model large macromolecular, or process high-resolution time-resolved structural determination often require high-performance computing clusters equipped with specialized hardware like GPUs.^20,27,33,37^

### B. The reproducibility challenge in computational science

The broader scientific community faces an ongoing challenge with research reproducibility, echoing concerns about the robustness of scientific conclusions dating back centuries. A survey highlighted in *Nature* involving 1576 researchers revealed significant difficulties, with over 70% failing to reproduce another scientist's experiments and 52% unable to reproduce their own.^4^ Increasing the complexity of computational workflows and datasets, in fields such as structural dynamics, has the potential to magnify these issues unless they are addressed.

For a given dataset (or datasets), the issue of reproducibility can be viewed from two perspectives: computational and scientific. Computational reproducibility can be considered as the problem of running the analysis pipeline used by the original researchers and obtaining results that are identical (with the exception of incidental changes such as timestamps, user or system names that may be embedded in output files, or processing logs by the program). There are many challenges to computational reproducibility: access to original inputs and parameters used for processing, and the ability to execute all programs (and their dependencies) at the same versions used by the original researchers. Scientific reproducibility is more challenging in some ways while simpler in others. The objective is to obtain a result of computational analysis that supports the same scientific conclusions, rather than byte-for-byte identical output files. In principle, alternative implementations of equivalent algorithms would identify errors undetected by purely computational reproducibility. Although this allows more flexibility for the computational tools used, it has more strict requirements for standardization and interoperability of file formats and algorithmic parameters.

Scientific research today depends heavily on computational methods—for data acquisition, management, analysis, modeling, simulation, and visualization. Computational reproducibility underpins replicability in science.^24^ Many seemingly irreproducible scientific findings stem from opaque computational workflows. Ensuring reproducible analysis workflows is, thus, a necessary (though not always sufficient) condition for confident, generalizable scientific knowledge. In research computing, these issues are often exacerbated by the complexity of the required software environments. Factors such as inconsistencies in software versions, underlying libraries, operating systems, hardware configurations, and compiler options can lead to different results even when using nominally identical methods.^14,24^ Furthermore, lack of access to primary experimental data and the specific software versions used for original analyses hinder verification and reuse.^21^ Managing the intricate web of software dependencies is a significant burden, potentially consuming substantial researcher time (up to 30% in computational projects) that could otherwise be spent on scientific inquiry.^15,18^ This “reproducibility crisis,”^4^ compounded by the inherent complexity of computational workflows, presents a critical barrier to ensuring the robustness and reliability of findings, particularly in data-intensive fields like structural dynamics.

### C. An integrated platform approach

Addressing these intertwined challenges of scientific complexity and reproducibility requires a holistic, multi-pronged approach. The SBGrid Consortium,^23^ established at Harvard Medical School, has developed a comprehensive research informatics platform built on three core pillars, evolving in response to community needs: standardized software access (Pillar 1), established over two decades; accessible primary data publication (Pillar 2), initiated in 2015; and scalable compute infrastructure (Pillar 3), representing our more recent focus driven by escalating computational demands and educational initiatives. This manuscript details these three components, illustrating how the SBGrid platform empowers researchers to tackle complex structural dynamics questions with enhanced rigor, efficiency, and speed by addressing computational friction through a consistent, reliable, and community-driven approach. Providing a stable software environment, facilitating access to published primary data, and offering scalable computation, SBGrid enables researchers to focus more effectively on the scientific challenges inherent in studying structural dynamics. We will first describe the SBGrid software collection and the Capsules environment that support its management^14^ (Pillar 1). Second, we will detail the SBGrid Data Bank (SBDB) for primary data publication^21^ (Pillar 2). Third, we will outline the SBCloud platform for scalable computation^20^ (Pillar 3). Finally, we will discuss the synergistic benefits arising from these functionally integrated components, their alignment with FAIR principles, and potential future directions relevant to advancing structural dynamics research.

## II. PILLAR 1: PROVIDING A CONSISTENT SOFTWARE ENVIRONMENT WITH CAPSULES

### A. The scientific software landscape in structural biology

Modern structural biology research is inextricably linked to a vast and diverse ecosystem of specialized software tools. This landscape includes potentially thousands or more applications^11,28^ spanning data processing, structure determination and refinement (e.g., CCP4,^3^ RELION,^31,32^ DIALS,^39^ Phenix,^19^ and BUSTER^7^), modeling and simulation (e.g., Amber,^8^ GROMACS,^2^ and NAMD^27^), visualization (e.g., VMD,^17^ PyMOL,^34^ Chimera/ChimeraX,^13,26^ and Coot^12^), and increasing leverage of artificial intelligence in new and existing tools.

SBGrid's strategy for adapting to rapidly evolving fields, such as AI/ML in structural biology, involves two key stages: discovery and integration. The discovery stage uses a dual approach of processing user requests alongside systematic monitoring of publication channels and code repositories. This allows us to identify key software, often within hours of its release. The subsequent integration stage is dedicated to overcoming the unique technical barriers presented by new software, which can range from complex build systems to specialized hardware requirements. The recent emergence of AI/ML tools in structural biology provides a prime example. For this class of software, the primary technical challenge is frequently its complex dependence on specific GPU hardware and CUDA libraries. It is here that our team's long-standing expertise in building and maintaining GPU-accelerated applications becomes crucial, enabling us to provide robust, ready-to-run versions of these tools. Recent examples of AI/ML software incorporated include AlphaFold,^41^ RoseTTAFold,^42^ DAQ,^43^ and DiffDock.^44^

These specialized software tools are developed globally, primarily within academic research groups, resulting in a heterogeneous collection with varied programming languages (C++, Python, Fortran, etc.), licensing models, distribution channels (websites, package managers, containers, and source code repositories), and complex, often unstated, dependencies on specific versions of libraries or other programs.^14^ For end-users, navigating this complexity, compiling source code, resolving conflicting dependencies, managing multiple versions, and ensuring compatibility with local operating systems [Linux, macOS, and Windows Subsystem for Linux (WSL2)] and hardware (including GPUs) represent a significant technical barrier and time sink. These challenges not only impede research efficiency but also fundamentally compromise computational reproducibility.^14^

### B. The SBGrid software collection

Since 2001, the SBGrid Consortium has worked to mitigate these software challenges for the structural biology community. SBGrid curates, installs, tests, and maintains a comprehensive collection of relevant scientific software, delivering it to member laboratories in a ready-to-run format. The collection has grown substantially over two decades and currently includes over 620 actively maintained software titles, encompassing more than 56 000 individual executables linked to over 510 000 dynamic libraries, requiring ∼2.3 TB for Linux and ∼0.8 TB for macOS installations.^14^ The collection spans crystallography, cryoEM, NMR, computational chemistry, visualization, and general utilities. Software is sourced globally, reflecting the community's development efforts and includes a mix of open-source (>220 titles) and academic/commercial licenses. The SBGrid curation model is inherently resource-intensive, requiring a dedicated technical team, but it provides tangible benefits by shifting the technical and operational burden away from individual research groups. In 2023, for example, we released 16 titles per month on average (12 updated versions and 4 new software titles), reflecting a scalable and sustained curation capacity across a large and growing collection.^14^ Additionally, a small team of fewer than five curators supports a community of over 5000 unique users each month, demonstrating the efficiency of this centralized service. By centralizing expertise, users can focus on research rather than maintenance. For newly developed titles or versions with cutting edge new features, the number of users requesting curation gives the curation team an additional signal to assist with prioritization.

### C. The capsules software execution environment

SBGrid developed the Capsules Software Execution Environment (CSEE) to address limitations of its original monolithic setup, which, while offering zero-configuration access, struggled with scalability and software conflicts as the collection expanded.^14^

Capsules and containers serve complementary roles in scientific computing workflows. SBGrid Capsules are optimized for managing the complex and dynamic interdependencies between applications within an integrated software collection, making them ideal for daily research and development.^14^ In contrast, containers excel at providing runtime isolation by packaging a single application together with its specific operating system dependencies into a portable unit. This makes them a powerful tool for distributing a self-contained application or ensuring bit-for-bit reproducibility of a final, frozen workflow. Importantly, these approaches are not mutually exclusive: the entire SBGrid software collection, or individual titles within it, can be readily incorporated into a container, allowing researchers to combine the flexibility of our curated environment with the encapsulation that containers provide.

Software within capsules is organized hierarchically by operating system, architecture, application title, and version. Central to the system are manually curated Environment Definition (ED) files, which specify environment variables, software dependencies, and platform- or version-specific logic necessary for correct execution. These ED files are written in the SBGrid Capsules Configuration Language, a domain-specific language enabling flexible and reproducible environment definitions.

During installation, SBGrid tools use ED files to generate lightweight runtime scripts called CR (Capsules Runtime) scripts. These scripts act as entry points for each executable, dynamically configuring the necessary environment before launching the software. By intercepting each command, a CR script ensures the right version, and its dependencies are isolated and properly initialized for each run. When multiple packages provide executables with the same name, the system uses a component called the Capsules Executable Mapper to determine which version to execute based on user, site, or system-level preferences.

Users activate the SBGrid environment through a single shell command and may optionally define preferred software versions in a personal configuration file. Capsules also address system limitations, such as path length, by consolidating all runtime scripts into a centralized directory. Altogether, Capsules streamline software access, maximize reproducibility, and simplify managing a diverse and evolving library of scientific applications.

### D. Relevance to structural dynamics

The stability, consistency, and ease of use provided by the Capsules environment directly benefit structural dynamics research, which often requires computationally intensive and complex workflows. One major advantage is robust workflow execution. Dynamics studies frequently involve chaining multiple software tools, such as when processing large datasets from time-resolved serial crystallography or cryoEM movie data. These pipelines often rely on tools like DIALS,^39^ RELION,^31,32^ or cryoSPARC.^29^ Similarly, analyzing long molecular dynamics trajectories may involve CPPTRAJ from Amber,^8^ MDAnalysis,^22^ clustering algorithms,^9,25^ or Markov State Models.^9^ Capsules ensure each step runs in the correct, isolated environment, minimizing failures due to library conflicts or mismatched versions. This improves workflow reliability and reduces time lost to debugging, which is essential for demanding analyses.

Simplified access to modern tools is another key benefit. Structural dynamics researchers often rely on the latest algorithms and specialized software, including tools for anisotropic diffuse-scattering analysis,^36^ multi-state refinement, or 3D variability analysis in cryoEM.^10,30^ For example, solutions such as cryoSPARC,^29^ RELION,^31,32^ CryoDRGN,^40^ and Scipion^1^ are essential for studying conformational heterogeneity in cryoEM. Capsules make these tools immediately usable without complex installations.

Crucially, the user-defined software version control feature allows researchers to repeat simulations, validate time-resolved data analyses, and perform consistent evaluations of conformational ensembles—supporting scientific rigor in structural dynamics studies.

## III. PILLAR 2: ENSURING ACCESS TO PRIMARY EXPERIMENTAL DATA VIA THE SBGRID DATA BANK

In response to community feedback recognizing the critical need for preserving and publishing the primary data, the SBGrid Databank (SBDB) launched in 2015^21^ as a data repository focused on primary x-ray diffraction datasets while supporting datasets for other techniques with similar characteristics. For macromolecular single-crystal monochromatic x-ray diffraction experiments, the primary dataset is typically composed of one or more sets of diffraction images, with a typical size range of ∼1 to ∼20 GB. Primary datasets, regardless of experimental technique, are originally recorded instrument data. The unchanging nature of these datasets distinguishes them from intermediate datasets and the structural models derived from these datasets that support drawing scientific conclusions.

Experimental metadata is centrally coordinated through data.sbgrid.org, with links to related datasets (such as Protein Data Bank^6^ entries for final models and intermediate data), EM Data Bank^35^ entries (where appropriate), and manuscripts describing the use of these datasets. These metadata links reduce the administrative burden on dataset depositors to duplicate metadata entries. Primary metadata is made available through a variety of standardized formats (json-ld, datacite) and propagated to the DOI system upon the public release of datasets. Support for replication of dataset files to other distribution sites or HPC facilities has been present since launch, as has support for direct computation on released datasets. User-verifiable dataset integrity is supported throughout the dataset lifecycle, and backend repository automation ensures the released data files match those on the depositor's storage. Dataset replication to the HMS HPC cluster allows for automated analysis pipelines to be run at a large scale on all released datasets. Between 2015 and the present, ∼859 datasets have been published through SBDB.

Several aspects of the SBDB collection and design are relevant to the field of structural dynamics and scientific reproducibility. Datasets for diffuse-scattering experiments (doi: 10.15785/SBGRID/201, doi: 10.15785/SBGRID/747, and doi: 10.15785/SBGRID/958) and molecular dynamics (doi: 10.15785/SBGRID/190) published through SBDB are directly applicable to the discipline. Two particular repository functions applicable to structural dynamics are: (1) the capability to support datasets with large storage requirements (such as time-resolved crystallography or cryoEM), when supported by sufficient infrastructure; (2) support for propagating machine-readable information on the relationships between datasets, including datasets of distinct types, to support integrative techniques.

## IV. PILLAR 3: PROVIDING SCALABLE COMPUTATION WITH SBCLOUD INFRASTRUCTURE

Complementing established software curation (Pillar 1) and data publication (Pillar 2), the most recent phase of SBGrid's development addresses the escalating need for scalable and accessible computational resources through the SBCloud platform. Modern structural biology is increasingly defined by data-intensive and computationally complex workflows. Techniques such as cryo-electron microscopy (cryoEM), x-ray free-electron lasers (XFEL), and molecular dynamics simulations generate large volumes of data and require substantial compute resources—often including GPUs, high-memory instances, and high-performance parallel storage.^20^ These demands exceed the capacity of many on-premise academic clusters, which are frequently oversubscribed, inconsistently maintained, or lacking key software and hardware support. As a result, researchers face significant delays, friction in reproducing results, and challenges scaling their analyses to accommodate new types of time-resolved or high-throughput experiments.

SBCloud's capabilities address these challenges by offering a managed, cloud-based computing environment tailored to the needs of structural biology and structural dynamics researchers. Built on AWS and maintained by the SBGrid Consortium, SBCloud provides a reproducible and scalable HPC platform.

**FIG. 1.**
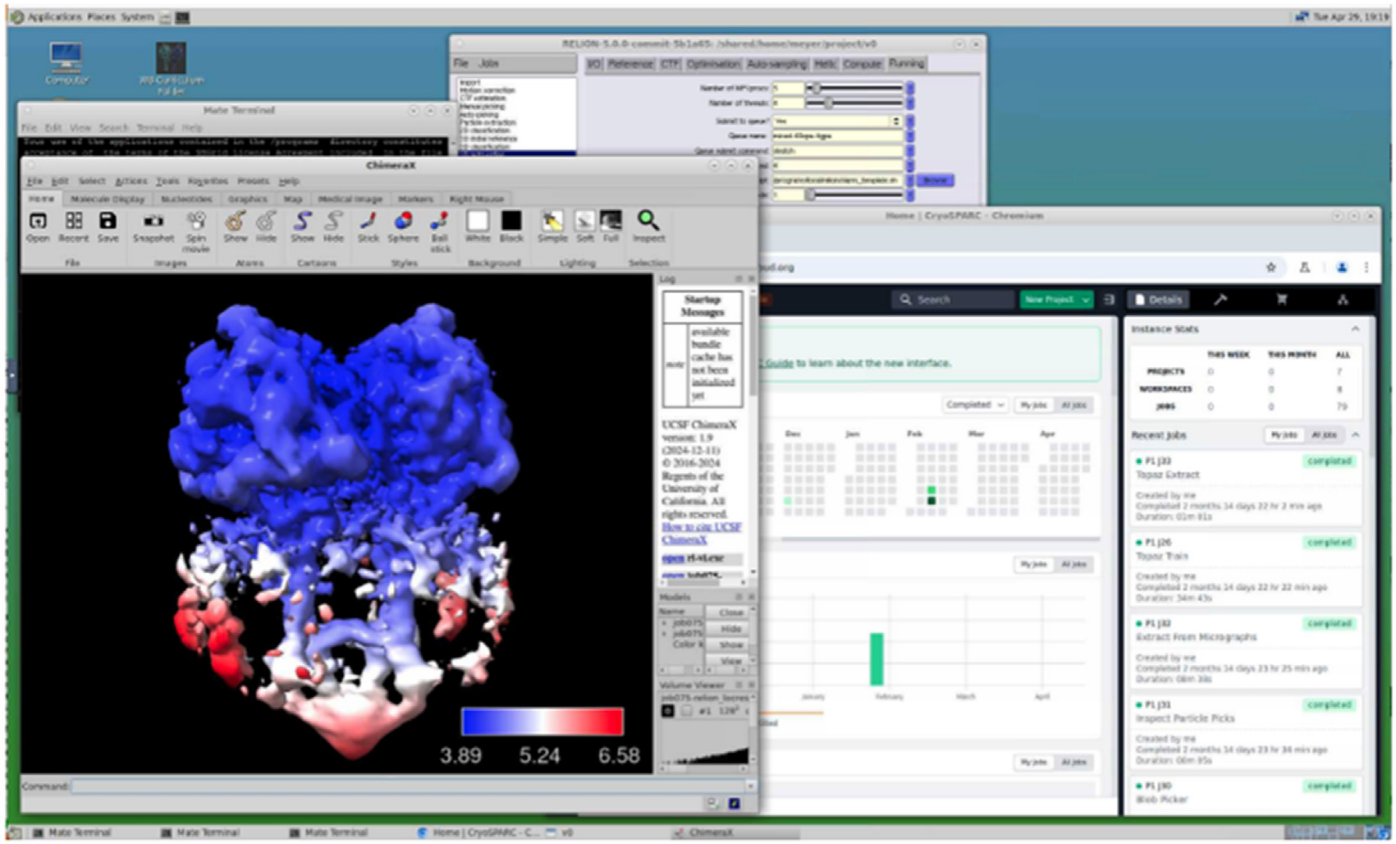
SBCloud virtual GPU desktop environment accessed via Open OnDemand, showcasing concurrent execution of ChimeraX, RELION 5, and cryoSPARC.

Each SBCloud deployment includes a Slurm-based cluster with NFS and Lustre based POSIX storage for various workloads and the SBGrid scientific software stack for analysis. Researchers interact with the system through Open OnDemand^16^—a browser-based interface that offers terminal access, graphical desktops, file browsers, and job submission tools familiar to users of academic HPC clusters (Fig. 1). Researchers can dispatch interactive or batch jobs to ephemeral compute nodes that are automatically scaled to match demand. Underlying resources—including instance types, storage configurations, and regional preferences—can be customized to meet specific scientific workflows.

Data transfer is supported through familiar, reliable tools such as Globus and rsync, allowing researchers to move data easily between institutional systems and SBCloud environments. These options reduce the barrier to entry for users by integrating into existing workflows and enabling efficient handling of large datasets. Each SBCloud deployment is defined through a consistent infrastructure-as-code model, ensuring that environments are version-controlled, reproducible, and aligned with best practices for secure, auditable research computing. By abstracting the complexity of cloud resource provisioning and system administration, SBCloud enables researchers to focus on their science rather than system maintenance.

**FIG. 2.**
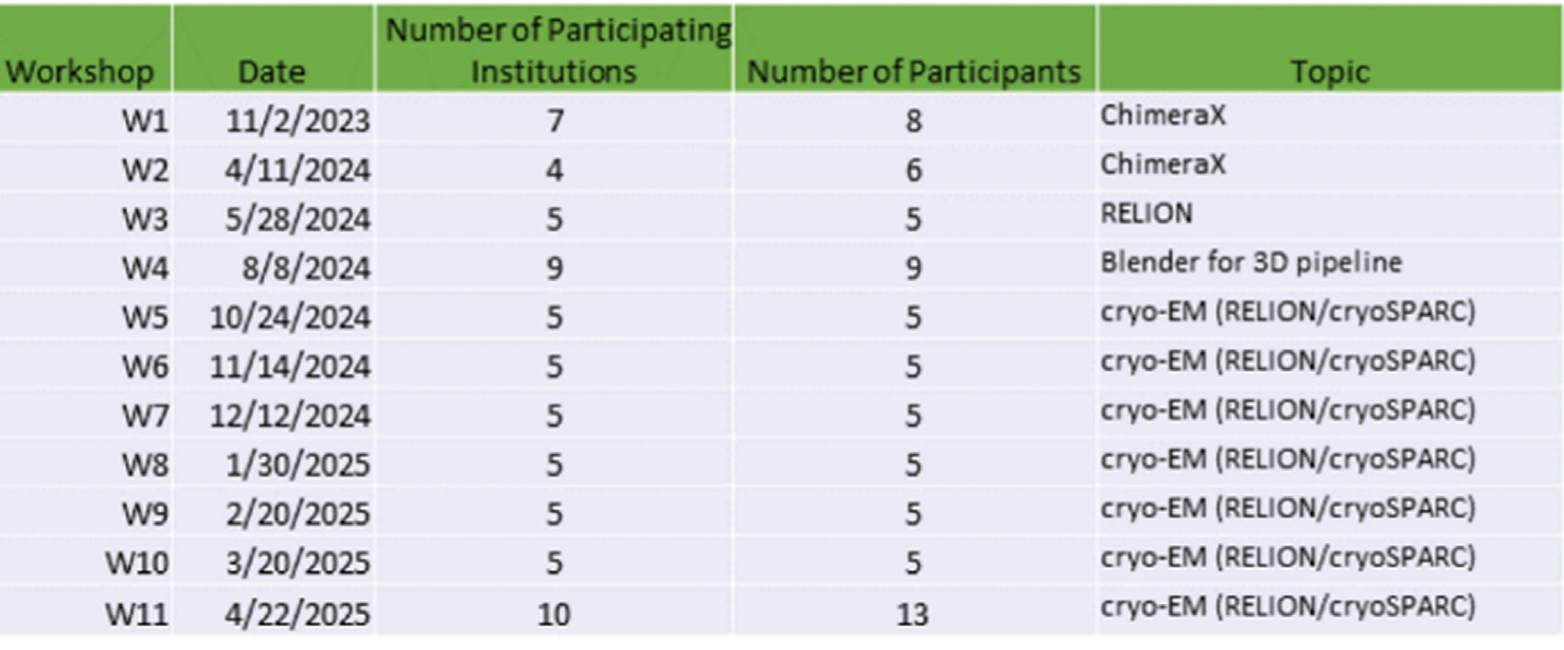
SBCloud-powered NIH R25 workshops: participation and timeline (November 2023–April 2025).

A motivating factor for the development of SBCloud was to support use in education and training, particularly through SBGrid's NIH R25-funded “Continuing Education for Structural Biology Mentors” program. These workshops leverage SBCloud to provide hands-on experience with introductory but realistic workflows. For instance, participants successfully processed a real-world cryoEM dataset (EMPIAR-11422) using a single-particle workflow focusing on RELION 5^31,32^ and cryoSPARC,^29^ demonstrating that SBCloud handles demanding analyses within a training context. From November 2023 to April 2025, SBCloud-powered 11 workshops, including international sessions, training participants from numerous institutions worldwide (Fig. 2). Particularly relevant for workshops with participants from diverse institutions, SBCloud provided all attendees with a uniform computing environment to learn and explore new workflows. This successful application in training underscores SBCloud's robustness, scalability, and ease of use, lowering barriers to entry for advanced computational techniques crucial for fields like structural dynamics.

By providing a consistent runtime environment for executing complex software pipelines, managing high-volume datasets, and enabling collaboration across institutions, SBCloud ensures computational workflows are reproducible and portable across deployments.

## V. DISCUSSION

### A. Functional integration and synergy of the SBGrid platform components

While each pillar of the SBGrid platform—Software, Infrastructure, and Data—offers independent utility, their conceptual synergy magnifies their leverage within a unified organizational framework. Direct technical integration varies among the components—the SBGrid software collection and SBCloud are tightly interwoven, whereas the SBGrid Data Bank (SBDB) serves as a functionally complementary data resource—creating a robust, comprehensive ecosystem capable of supporting structural dynamics research. Researchers benefit immensely from access to a vast library of reliable, version-controlled software supported by Capsules, the capacity to perform demanding computations on scalable resources, and access to citable primary experimental data via SBDB. This functional integration minimizes friction, maximizes research efficiency, and accelerates the path from data to dynamic insight.

The primary benefit of our centralized model is the significant efficiency it creates across the research ecosystem. While the professional curation practiced by SBGrid is resource-intensive for our team, it obviates the need for this complex work to be duplicated inefficiently at every member institution or individual laboratory. This efficiency, as discussed in Sec. II B, is exemplified by a small team of fewer than five curators supporting a community of over 5000 unique monthly users. This “curate once, deploy to many” approach enables rapid, coordinated updates (e.g., 16 new or updated titles per month in 2023), expert support, and consistent software behavior and performance across all member sites. In contrast, fully decentralized or community-driven packaging can lead to inconsistent support and build reliability, shifting the maintenance burden onto end-users and away from their primary research objectives.

The SBGrid environment is targeted toward broad portability and minimal end-user setup. For research projects where software development itself is a significant component of the project, such as creating new methods or performing workflow-profiled, hardware-specific optimizations, a dedicated, local development environment is often more suitable.

The SBGrid service model is staffed by a dedicated, multidisciplinary team. While the ongoing requirements for curation, integration, and support are significant, this centralized investment lessens the technical, logistical, and labor overhead for our global user base, allowing community members to focus on research rather than software maintenance.

### B. Alignment with FAIR principles and reproducibility

The SBGrid platform aligns with core aspects of the FAIR principles for research software (FAIR4RS)^5,14,38^ and scientific data,^21^ with each pillar contributing distinctly. It contributes to computational reproducibility by providing a stable, version-controlled software environment via our curated software application collection (Pillar 1). Pillar 2 (Data) promotes Findability, Accessibility, and Reusability through the SBGrid Data Bank, which assigns DOIs, exposes machine-readable metadata, and links datasets to related publications and models. Interoperability is improving with structured metadata and dataset relationships but varies by data type.

While the software application collection (Pillar 1) and accessible data (Pillar 2) provide a critical foundation, end-to-end reproducibility requires additional measures. Our platform supports this through two primary modalities. For flexibility, users can integrate the SBGrid software collection into external tools like containers to build a portable and reproducible workflow. Alternatively, for users seeking a fully integrated solution, the infrastructure-as-code capabilities of SBCloud (Pillar 3) offer a direct path to a provisioned, reproducible compute environment by combining the software stack with a defined hardware and operating system configuration. For a fully reproducible workflow, the relevant metadata for this environment, and the fully scripted workflow, would need to be treated like any other scholarly object (deposited in a FAIR repository) and linked with input data similarly deposited.

## VI. CONCLUSION

SBGrid's core value lies in its integrated service model, which combines technical infrastructure with professional curation, ongoing user support, and institutional licensing management. This approach offers distinct advantages for multi-user environments such as university core facilities, institutional research clusters, and international training workshops, where stable and uniformly supported software is essential. It also empowers individual research groups, particularly those lacking dedicated IT support, by shifting the significant operational burden of software management away from scientific personnel and onto a professional team. Furthermore, this service model benefits the academic software developers themselves. Additionally, this service model provides critical scientific stewardship. Our approach can extend the useful lifetime of valuable but unmaintained software, and in some cases, SBGrid serves as the sole archival source for legacy tools. By centralizing the complex tasks of multi-platform packaging, distribution, and front-line user support, we enable developers to focus on innovation and core scientific features, thereby accelerating progress across the entire field.

These components reduce computational friction and simplify incidental complexity, freeing researchers to focus on interpreting molecular motions and modeling structural transitions. By reducing friction and enabling a focused scientific process, the SBGrid platform establishes a foundation adaptable to common challenges across computational structural biology and related fields.

## Data Availability

Data sharing is not applicable to this article as no new data were created or analyzed in this study.
